# Multiple imputation for an incomplete covariate that is a ratio

**DOI:** 10.1002/sim.5935

**Published:** 2013-08-06

**Authors:** Tim P Morris, Ian R White, Patrick Royston, Shaun R Seaman, Angela M Wood

**Affiliations:** a Hub for Trials Methodology Research, MRC Clinical Trials UnitAviation House, 125 Kingsway, London WC2B 6NH, U.K.; b MRC Biostatistics Unit, Institute of Public HealthRobinson Way, Cambridge CB2 0SR, U.K.; c Department of Public Health & Primary Care, Strangeways Research Laboratory2 Worts Causeway, Cambridge CB1 8RN, U.K.

**Keywords:** missing data, multiple imputation, ratios, compatibility

## Abstract

We are concerned with multiple imputation of the ratio of two variables, which is to be used as a covariate in a regression analysis. If the numerator and denominator are not missing simultaneously, it seems sensible to make use of the observed variable in the imputation model. One such strategy is to impute missing values for the numerator and denominator, or the log-transformed numerator and denominator, and then calculate the ratio of interest; we call this ‘passive’ imputation. Alternatively, missing ratio values might be imputed directly, with or without the numerator and/or the denominator in the imputation model; we call this ‘active’ imputation. In two motivating datasets, one involving body mass index as a covariate and the other involving the ratio of total to high-density lipoprotein cholesterol, we assess the sensitivity of results to the choice of imputation model and, as an alternative, explore fully Bayesian joint models for the outcome and incomplete ratio. Fully Bayesian approaches using Winbugs were unusable in both datasets because of computational problems. In our first dataset, multiple imputation results are similar regardless of the imputation model; in the second, results are sensitive to the choice of imputation model. Sensitivity depends strongly on the coefficient of variation of the ratio's denominator. A simulation study demonstrates that passive imputation without transformation is risky because it can lead to downward bias when the coefficient of variation of the ratio's denominator is larger than about 0.1. Active imputation or passive imputation after log-transformation is preferable. © 2013 The Authors. Statistics in Medicine published by John Wiley & Sons, Ltd.

## 1 Introduction

Missing values of covariates are a common problem in regression analyses. Missing data are classified as being *missing completely at random* (MCAR) if missingness does not depend on observed or unobserved data, *missing at random* (MAR) if missingness does not depend on unobserved data given observed data, or *missing not at random* if missingness depends on missing data even given the observed data 1. Amongst methods that attempt to deal with missing data, rather than discarding them, multiple imputation (MI) can provide valid inference under MAR and has become popular in practice since its inception over 30 years ago 2.

Briefly, MI works as follows. Missing values are replaced with imputed values, drawn from their posterior predictive distribution under a model given the observed data. We term this model the *imputation model*. The process is repeated *M* > 1 times, giving *M* imputed datasets with no missing values. Each imputed dataset is analysed using the model that would have been used had the missing values been observed. We call this model the *analysis model*. The *M* estimates of each parameter of interest are then combined using ‘Rubin's rules’ 3. When the imputation model is correctly specified, Rubin's rules can provide standard errors and confidence intervals that fully incorporate uncertainty due to missing data.

MI is an attractive tool for analyses with missing data: The nuisance issue of modelling missing data is neatly separated from the analyses of substantive interest; the imputation model can make use of auxiliary variables that it would be undesirable to include as covariates in the analysis model (such as post-baseline measurements in a randomised controlled trial); the same *M* imputed datasets can be used for a variety of substantive analyses; and the imputation model can be tailored to reflect possible departures from MAR, which is helpful for sensitivity analysis.

Ratios are commonly used as covariates in regression analyses; examples are body mass index (BMI = Weight in kg ÷ (Height in m) ^2^) 4, waist–hip ratio 5, urinary albumin-to-creatinine ratio (Albumin concentration in mg/g ÷ Creatinine concentration in mg/g) 6, and what we refer to as ‘cholesterol ratio’ (Total cholesterol in mg/dL ÷ HDL in mg/dL) 7.

An individual's ratio measurement may be missing for one of the three reasons:

1. The denominator is missing.
2. The numerator is missing.
3. Both components are missing.

For both 1 and 2, the ratio is semi-missing rather than fully missing; that is, one of the two components is observed. Ratio missingness due to more than one of these reasons for different observations in the same dataset means it is not obvious how best to impute the ratio. A mixture of reasons 1 and 2 is particularly awkward.

One reasonable question at this stage is, ‘Why use a ratio covariate?’ There are mathematical arguments against their use 8. Senn and Julious claim that ratios are always poor candidates for parametric analysis unless the components, and therefore the ratio, follow a lognormal distribution or the ratio's coefficient of variation (CV) is small 9. We make three points. First, applied researchers *do* use ratios, and we are unlikely to persuade them to stop, especially because the use of certain ratios is well established; we should be pragmatic and try to guide practitioners on how to analyse datasets involving incomplete ratio covariates. Second, arguments against ratios assume that a ratio is not the correct functional form for a covariate, but it may be. Third, ratios are not used by accident: A ratio may be of genuine substantive interest when its separate components are not. For example, BMI is widely used because it measures weight-for-height and as such is regarded as a proxy measure of body fat. Substantive interest is in the influence of body fat on outcome, not weight or height. Weight alone may be considered a measure of body fat, but BMI is measured with less error because it aims to remove the effect of height (although it may not do so completely or accurately). It is our opinion that when researchers propose a relationship they believe, such as the influence of a ratio on outcome, this should not be cast aside lightly. The substantive question should not be altered for statistical convenience unless we have little choice.

We assume that the aims of analysis are unbiased estimation of a parameter describing the association between a ratio and some outcome, confidence intervals with the ascribed coverage and fully efficient parameter estimation. There may be other covariates in the analysis model, and primary interest may be in one of these, but the properties of the ratio parameter estimator are important nonetheless. There has been no previous methodological work on MI for a ratio covariate, although White *et al.* 10 and Bartlett *et al.* 11 allude to the issue, but practitioners are imputing ratio covariates nonetheless 12. We aim to highlight issues with imputing an incomplete ratio covariate and to identify imputation strategies that are practicable for applied statisticians.

Despite the positive features listed previously, MI is neither the only approach to dealing with missing covariates, nor necessarily the best approach for any given analysis. Joint models for the outcome and covariates may be superior because they make use of the full likelihood in a coherent way. In this paper, we also investigate results for fully Bayesian joint models.

The remainder of this paper is as follows. In Section 2, we introduce and describe our two motivating datasets; in Section 3, we consider candidate models for imputing incomplete ratios. Section 4 presents two case studies, contrasting the different imputation models (for the datasets introduced in Section 2). Section 5 presents a simulation study in a simpler setting than our case studies; and Section 6 is a discussion.

## 2 Datasets: *Aurum* and *EPIC*-Norfolk

For both of our datasets, regression analyses involving a ratio as a covariate have previously been published 4,7. The analysis models used in our example analyses are not the same as the original articles because of the following: (i) we want to keep the analysis models and imputation models relatively simple, and (ii) we do not wish to make any substantive claims about these data. Therefore, we have chosen to use analysis models resembling but not matching those used in the earlier publications 4,7.

For both datasets, the analysis model is the Cox model,


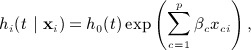
(1)

where *H*_0_(*t*) is the nonparametric baseline hazard function at time *t*, *h*_*i*_(*t * | * ***x**_*i*_) is the hazard for the *i*th individual and *x*_*ci*_ is the value of the *c*th covariate in the *i*th individual. Survival (or censoring) times are assumed to be fully observed.

### 2.1 The *Aurum* cohort

The *Aurum* dataset comes from a South African cohort study of 1350 HIV-infected participants starting antiretroviral therapy. Participants were recruited from 27 centres in five provinces between February 2005 and June 2006 and followed to March 2007. Information was recorded on a range of baseline characteristics, and participants were followed up for death. The aim of the work by Russell *et al.* 4 was to estimate the influence of hæmoglobin on mortality using a Cox model. Of the participants, 1348 had a recorded time of death/censoring, with 185 deaths occurring within the follow-up time. We restrict our analysis to these 1348 individuals.

The analysis model is (1) with *p* = 6, where *x*_1_, … ,*x*_6_ are age in years, sex, hæmoglobin in g/mL, viral load in copies per mL, CD4 count in cells per *μ*L and BMI. Table 1 provides a summary of these covariates and of weight and height. We give transformations of the covariates used in the analysis model, and summarise the transformed measure in the final column. Note that 381 (28%) patients are missing a weight and/or height measurement, but only five of these have height missing when weight is observed. Five of the covariates are continuous, and one (sex, which is complete) is categorical. Hæmoglobin, weight, height ^2^ and BMI appear to be approximately normal on the transformed scale, while (log) viral load and (square root of) CD4 count do not. We focus on the estimation of *β*_3_ and *β*_6_, the log hazard ratios for hæmoglobin and BMI, respectively, (hæmoglobin was the focus of the original publication 4).

**Table 1:** *Aurum* summary of covariates and of the analysis model and components of body mass index (BMI); *n* = 1348.

	Covariate	Frequency missing (%)	Mean (SD) or frequency (%)
*x*_1_	Age (years)	0 (0%)	37 (9)
*x*_2_	Sex: male	0 (0%)	542 (40%)
*x*_3_	Hæmoglobin (g/mL)	143 (11%)	11.4 (2.3)
*x*_4_	*Viral load (copies per mL)	162 (12%)	4.8 (0.8)†
*x*_5_	*CD4 count (cells per *μ*L)	94 (7%)	8.9 (4.5)†
*x*_6_ = *a*_1_ / *a*_2_	BMI (kg/m ^2^)	381 (28%)	21.9 (4.9)
*a*_1_	‡Weight (kg)	376 (28%)	58 (12)
*a*_2_	‡Height (m ^2^)	275 (20%)	2.7 (0.3)†

* Transformation used for viral load is log _10_(*x*_4_); transformation used for CD4 count is 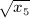. These are standard transformations in HIV research, and we use them in the imputation models and the analysis models.

† †Summarised on transformed scale.

‡ ‡Only enters into the analysis model via BMI.

### 2.2 The *EPIC*-Norfolk cohort

The *European Prospective Investigation Into Cancer and Nutrition* (EPIC)-Norfolk study is a large cohort study designed to investigate the link between dietary factors and cancer. Dietary and non-dietary factors were collected at baseline, and participants were followed up for cancer and non-cancer outcomes. We use some of the non-dietary characteristics as covariates and time to death as the outcome.

The analysis model is (1) with *p* = 6, where *x*_1_, … ,*x*_6_ are age, sex, smoking status, systolic blood pressure, diastolic blood pressure and cholesterol ratio. We summarise these six covariates and total cholesterol and HDL in Table 2; none are transformed. In total, 2155 (9%) participants are missing a total cholesterol and/or HDL measurement. Total cholesterol is always missing when HDL is missing. Incomplete covariates are all continuous and appear approximately normal, except for HDL, which is positively skewed. We focus on the estimation of *β*_6_, the log hazard ratio for cholesterol ratio.

**Table 2:** *EPIC*-Norfolk summary of covariates of the analysis model and of components of cholesterol ratio; *n* = 22 754.

	Covariate	Frequency missing (%)	Mean (SD) or frequency (%)
*x*_1_	Age (years)	0 (0%)	59 (9)
*x*_2_	Sex: male	0 (0%)	10145 (45%)
*x*_3_	Smoking status: ever smoked	0 (0%)	11971 (53%)
*x*_4_	Systolic blood pressure (mm Hg)	52 (<1%)	135 (18)
*x*_5_	Diastolic blood pressure (mm Hg)	52 (<1%)	82 (11)
*x*_6_ = *a*_1_ / *a*_2_	Cholesterol ratio	2155 (9%)	4.7 (1.6)
*a*_1_	†Total cholesterol (mg/dl)	1514 (7%)	6.2 (1.2)
*a*_2_	†HDL (mg/dl)	2155 (9%)	1.4 (0.4)

† †Only enters into the analysis model via cholesterol ratio.

## 3 Methods and models

### 3.1 Model for analysis

The analysis model is the Cox model (1) with *p* covariates (*x*_1_, … ,*x*_*p*_) made up of the ratio *x*_*p*_ = *a*_1_ / *a*_2_ and *p* − 1 other covariates (*x*_1_, … ,*x*_*p* − 1_), which we denote (**z**,**w**) where **z** are incomplete and **w** are complete (in both example datasets, we have **z** and ** w**).

### 3.2 Models for missing data

We list candidate models for the covariates in Table 3 (note the *Label* column, which we henceforth use to refer to models). For MI, the outcome must be explicitly included as a covariate in the imputation model 13. In Table 3, we denote outcome by *f*(*y*_*i*_). For the Cox model, *f*(*y*_*i*_) involves a censoring indicator and the Nelson–Aalen estimate of the cumulative hazard function to the survival time (an approximation to the cumulative baseline hazard function *H*_0_(*t*) 14), included as separate covariates in the imputation model. When the analysis model is linear or logistic regression, *f*(*y*_*i*_) = *y*_*i*_.

**Table 3:** Candidate imputation models for x_*i*_.

Imputation model	Label	Relationship to analysis model
(**z**_*i*_,*x*_*pi*_* * | * f*(*y*_*i*_),**w**_*i*_) ∼ MVN	M1	Compatible
(**z**_*i*_,*x*_*pi*_,*a*_1*i*_* * | * f*(*y*_*i*_),**w**_*i*_) ∼ MVN	M2	Semi-compatible
(**z**_*i*_,*x*_*pi*_,*a*_2*i*_* * | * f*(*y*_*i*_),**w**_*i*_) ∼ MVN	M3	Semi-compatible
(**z**_*i*_,*x*_*pi*_,*a*_1*i*_,*a*_2*i*_* * | * f*(*y*_*i*_),**w**_*i*_) ∼ MVN	M4	Semi-compatible
†(**z**_*i*_,*a*_1*i*_,*a*_2*i*_* * | * f*(*y*_*i*_),**w**_*i*_) ∼ MVN	M5	Incompatible
‡(**z**_*i*_,ln(*a*_1*i*_),ln(*a*_2*i*_)* * | * f*(*y*_*i*_),**w**_*i*_) ∼ MVN	M6	Incompatible

† †Passive imputation of 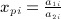 is required.

‡ ‡Passive imputation of *x*_*pi*_ = exp[ln(*a*_1*i*_) − ln(*a*_2*i*_)] is required.

### 3.3 Compatibility in relation to active and passive imputation

Multiple imputation can provide an approximation to fitting a joint model if the models for imputation and analysis are compatible 15, where a joint model may be either maximum likelihood or Bayesian (if the joint model is Bayesian, compatibility also requires that priors are non-zero over the entire parameter space). Considering whether or not the models M1–M6 are compatible with the analysis model helps us to formulate hypotheses and understand future results.

By ‘compatible’, we mean that a joint model exists that implies both the imputation model and the analysis model as conditional models. This does not mean that the joint model is correct, but that the analysis model and imputation model are both implied by it, and so the MI procedure is coherent. Appendix A describes how to tell if models are compatible and works through two examples of imputation models where one is compatible and the other is not (A.1 and A.2, respectively).

Compatibility is related the concept of ‘congeniality’, and the term congeniality is often used to mean compatibility 16,17,10. Congeniality requires that the joint model from which the imputation and analysis models can be derived is Bayesian. Further, the researcher's incomplete and complete data procedures must be specified, and the inferences must be asymptotically equivalent to a Bayesian model. We refer interested readers to Meng 18.

Non-compatibility of models is not always problematic; Meng 18 and Rubin 19 have both shown that there can be some *benefit* to using imputation models that correctly draw on information not used by the analysis model. Collins, Schafer and Kam demonstrate via simulation that auxiliary variables (i.e. variables that are in the imputation model but not the analysis model) are unlikely to be harmful, and may be of benefit by making the MAR assumption more plausible, while ‘restrictive’ imputation strategies can lead to problems 20.

We therefore distinguish between two types of non-compatibility: If there is a special case of the imputation model that is compatible with the analysis model, as when it includes auxiliary variables, then the imputation model is termed ‘semi-compatible’ (following Liu *et al.* 21); otherwise, the imputation model is simply termed ‘incompatible’. In previous work, imputation models that are compatible or semi-compatible appear to perform well even when misspecified 22,23, but this is not necessarily true for imputation models that are incompatible 23,20. We hypothesise that imputation models that are compatible or semi-compatible will be more robust to modest degrees of misspecification than models that are incompatible.

Imputation of a ratio is performed either actively or passively. Of the imputation models listed in Table 3, only M1 is compatible with the analysis model. Of the remaining models M2–M4, which use active imputation, are semi-compatible because they include *a*_1_ and/or *a*_2_, which do not appear in the analysis model, as auxiliary variables in the imputation model; models M5 and M6, which use passive imputation, are incompatible with the analysis model because *x*_*p*_ is present in the analysis model but not in the imputation model, while *a*_1_ and *a*_2_ are present in the imputation model but not in the analysis model. We expect models M5 and M6 to be prone to bias and poor coverage, despite making use of all the observed data when imputing the ratio.

### 3.4 Motivation for missing data models

The choice of a model listed in Table 3 might be motivated by the way it makes use of observed information in *a*_1_,*a*_2_, which will depend on the pattern of missingness.

Model M1 may be a good approach when *a*_1_,*a*_2_ are missing simultaneously. If *a*_1_ is only missing when *a*_2_ is missing, M2 may be used because model M2 makes use of observed *a*_1_ values when imputing the ratio, and there is no information in *a*_2_ about missing values of *a*_1_ that might be used to improve imputation of *x*_*p*_. (Conversely, if *a*_2_ is only missing when *a*_1_ is missing, M3 may be attractive.) Note that M2 and M3 do not respect the deterministic relationship *x*_*p*_ = *a*_1_ / *a*_2_.

Model M4 makes use of information on *a*_1_,*a*_2_ by imputing both alongside *x*_*p*_; this may be motivated by having *a*_1_, *a*_2_ or both missing. This is similar to the approach advocated by von Hippel 22, which has been termed *just another variable* 10,23. As with M2 and M3, the model ignores the deterministic relationship *x*_*p*_ = *a*_1_ / *a*_2_ and assumes multivariate normality. This will appear a bizarre assumption; it is clearly wrong because the distributions of two of these variables must define the distribution of the third, yet software does not know this and will sample without complaint. If the assumption made by M4 is uncomfortable, we may be attracted to M5 or M6.

Model M5 is incompatible with the analysis model (Appendix A.1) and requires *x*_*p*_ to be imputed passively from imputed values of *a*_1_ / *a*_2_. The components *a*_1_,*a*_2_ are not auxiliary but completely determine the values of *x*_*p*_. The ratio of *a*_1_ and *a*_2_, which are both normal, is expected to be heavy tailed.

M6 alters the problem by transforming *x*_*p*_ into a linear function of its logged components and passively imputing it. Model M6 guarantees that imputed values of *a*_1_,*a*_2_ are positive, as with all observed ratios. While this may be desirable, it is important to remember that our primary goal is valid inference, and we are not trying to recreate the missing values 19,24. The cosmetics of this model should therefore be a secondary consideration.

We have omitted from Table 3 the imputation model (**z**_*i*_,ln(*x*_*pi*_)* * | * f*(*y*_*i*_)) ∼ MVN. We do not consider this because ln(*x*_*p*_) = ln(*a*_1_) − ln(*a*_2_) where ln(*a*_1_) and ln(*a*_2_) are normal, and the sum of two normal distributions is normal. Model M6 is therefore equivalent to imputing ln(*x*_*p*_) but makes more use of the observed data when components are not simultaneously missing. The only setting where modelling ln(*x*_*p*_) alone is appropriate is if (*a*_1_,*a*_2_) are always either both observed or both missing. In this case, the model would then be equivalent to M6.

To summarise our discussion of the models in Table 3, there are conceptual problems with each one: Model M1 is compatible with the analysis model but does not use information on observed *a*_1_ or *a*_2_ when the other component is missing; M2–M4 are likely to be misspecified; and M5 and M6, the two models that make use of all the observed information on *a*_1_ and *a*_2_ and respect the relationship *x*_*p*_ = *a*_1_ / *a*_2_, are incompatible with the analysis model.

### 3.5 Software and details of imputation

We used Stata 12's misuite for MI in our case studies and simulations in Section 5 25,26. We performed multiple imputation using mi impute mvn, and implemented Rubin's rules using mi estimate.

Advice on the number of imputations typically suggests that a small number (fewer than 10) is sufficient 16. This idea comes from comparing the length of confidence intervals based on *M* imputations to intervals based on ∞ imputations. Our view on choosing the number of imputations, described in White *et al.* 10, is slightly different, being based on the reproducibility of analyses. To achieve negligible Monte Carlo error from our MI analyses, we use *M* = 300 imputations for the *Aurum* case study and *M* = 100 for *EPIC-Norfolk*. Note that we are not advocating such large values of *M* in general.

Our imputation models, all of which are based on a multivariate normal model, used a burn-in of 1000 iterations of the MCMC chain. Thereafter, we stored imputed datasets at every 10th iteration of the chain.

## 4 Case studies

This section presents the results for MI. However, in analyses with missing data, Bayesian models are widely regarded as a sensible alternative if there is reason to be suspicious of MI results. We outline and present Bayesian analyses of the *Aurum* and *Epic* datasets, corresponding to the MI approaches presented in this section, in Appendix B.

### 4.1 Imputing body mass index in the *Aurum* cohort

The MI procedures took between 2 min, 7 s (M1) and 2 min, 44 s (M6) to impute 300 times, fit the analysis model in each imputed dataset and use Rubin's rules to combine estimates.

**Figure 1:**
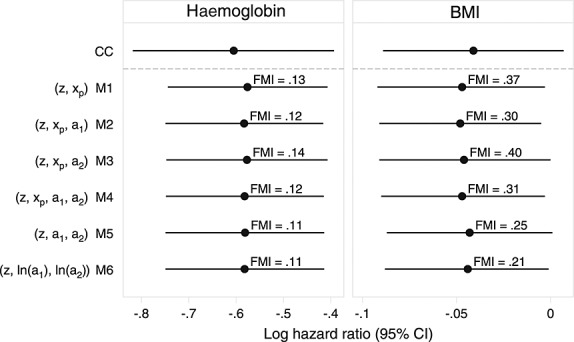
Results from analyses of *Aurum* data under different models for imputing body mass index (BMI). The estimated fraction of missing information (FMI) is given next to multiple imputation analyses.

Figure 1 shows estimates resulting from different imputation models. There is very little difference in the point estimates or width of confidence intervals; all returned essentially the same result. The number of imputations meant Monte Carlo error was negligible, at a maximum reaching 1/50th of the estimated standard error. The relative efficiency versus infinite *M* was > 0.999 for all models. For both hæmoglobin and BMI, the MI estimates gave a slight change in the point estimate and a small reduction in the width of confidence intervals as compared to complete cases.

### 4.2 Imputing cholesterol ratio in the *EPIC*-Norfolk cohort

For MI of the *EPIC*-Norfolk data, we used *M* = 100. We used a smaller number of imputations than in *Aurum* because only 9% of individuals were missing cholesterol ratio. MI took between 19 min, 2 s (M1) and 21 min, 0 s (M5) to impute 100 times, analyse each imputed dataset and combine estimates using Rubin's rules. The relative efficiency versus infinite *M* was > 0.999 for all models except M5, where relative efficiency was 0.991.

**Figure 2:**
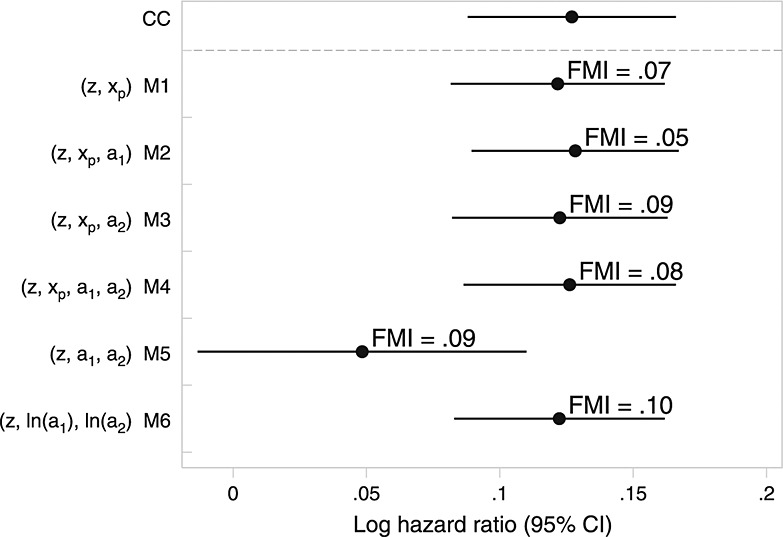
Results from analyses of *EPIC*-Norfolk data under different models for cholesterol ratio. The estimated fraction of missing information (FMI) is given next to multiple imputation analyses.

There was consistency between estimates from models that impute cholesterol ratio directly (Figure 2). Monte Carlo error for point estimates was negligible (around 0.0005, less than 1/50th of the standard error) for all models except M5 where it was 0.003. MI models are less consistent than in the Aurum MI analyses but would in five of six cases give similar substantive conclusions. These estimates are also very similar to complete-cases analysis and, interestingly, the imputation model that passively imputes cholesterol ratio through log-total cholesterol and log-HDL. However, the estimate after the standard passive imputation approach (M5) is much closer to the null, with wider confidence intervals.

**Figure 3:**
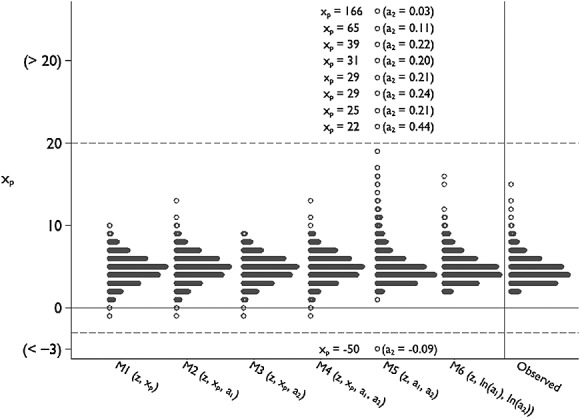
Dotplot of imputed cholesterol ratio for single (typical) imputed datasets in *EPIC*-Norfolk under models M1–M6. Imputed values of *x*_*p*_ < 3 or *x*_*p*_ > 20 are not plotted but represented according to rank; imputed values of (*x*_*p*_,*a*_1_) are listed.

Figure 3 demonstrates the problem with model M5 in the *EPIC*-Norfolk data, plotting imputed values of cholesterol ratio from a single, typical, imputed dataset under models M1–M6 alongside 2155 randomly selected observed values. The largest observed value of cholesterol ratio was 15.7. Note that for model M5, some imputed values were very large or very small; plotting these extreme values distorted the *y*-axis, and so we have censored the *y*-axis below − 3 and above +20, ranking and listing the values of imputed HDL and cholesterol ratio values outside of this range.

The problem with M5 arises because the mean and SD of HDL are 1.42 and 0.42, respectively, meaning its coefficient of variation (CV) is 0.30, resulting in a danger of *a*_2_ being imputed close to zero or even negatively. This CV is far larger than in the *Aurum* data, where CV(height ^2^) = 0.11 and imputed values are never close to zero (data not shown).

Figure 3 also highlights the difference between the other imputation models. Imputation on the log scale (M6) is the only model to guarantee that *a*_1_,*a*_2_ and *x*_*p*_ are positive. Further, the imputed values closely resemble the observed. M1–M4 can and did impute some *x*_*p*_ < 0; these models all assume *x*_*p*_ ∼ N, and so the distribution of imputed values is symmetrical about its mean. By looking at Figure 3, model M6 appears to be appealing, while from a statistical inference perspective (Figure 2), there appears to be little to choose between M6 and M1–M4. From all perspectives, M5 is a poor choice.

#### 4.2.1 Predictive mean matching

A natural question about model M5 that arises from Figure 3 is whether removing the high-leverage points could reduce the bias. For example, a truncated normal imputation model could be used to invoke the constraint *x*_*p*_ > 0, which would remove the negative outliers of model M5.

A better alternative, which can also remove the positive outliers, is *predictive mean matching* (PMM) 27,28,10. Briefly, the imputation model is fitted, and for each individual with a missing value, the *k* individuals (‘donors’) with observed values with the closest predicted mean are identified. One of these is selected at random and their value ‘donated’ as the imputed value. This ensures that imputed values are within the range of observed values.

To improve model M5, PMM is most easily implemented in a chained equations procedure 10. Imputation of *a*_1_ and *a*_2_ uses PMM, and *x*_*p*_ is passively imputed. The largest possible imputed value of *x*_*p*_ is then the ratio of the largest observed value of *a*_1_ to the smallest observed value of *a*_2_ (and vice versa for the smallest imputed value of *x*_*p*_).

We used this imputation model on the *EPIC*-Norfolk data, using *k* = 10 and storing imputed values after 10 cycles of chained equations. This reduced the bias of model M5, giving an estimated log-hazard ratio of 0.119 (95% CI 0.079–0.159). See Appendix C for the full results.

## 5 Simulation study

### 5.1 Design

We performed a simulation study designed to investigate models M1–M6 in a simpler setting than the two case studies. With *x*_*p*_ as the only covariate and a continuous outcome *y*, we investigated the performance of the imputation models and how this varied with the strength of *x*_*p*_–*y* association and the CV of the ratio's denominator, CV(*a*_2_). This affects the distribution of *x*_*p*_, and we hypothesise that when CV(*a*_2_) is large, model M5 will be biased. An imputed value of *a*_2*i*_ may be very small, meaning the corresponding value of *x*_*pi*_ will be large, and possibly outside the range of observed *x*_*p*_. The *x*_*pi*_ will thus have high leverage. For such values, there are unlikely to be appropriately large or small *y* to preserve the true *x*_*p*_–*y* relationship, which leads us to expect bias towards no association.

Scenarios investigated include two values of CV(*a*_2_): 0.1, taken from height ^2^ in the *Aurum* data, and 0.3, taken from HDL in the *EPIC*-Norfolk data; we vary these factorially with *R*^2^ values of 0.1 and 0.3. We performed all simulations using Stata 12 25. Our simulation procedures were as follows:

1. Simulate *n* = 500 complete values of ln(*a*_1_),ln(*a*_2_) to follow a bivariate normal distribution. In our first scenario, the mean, standard deviation and correlation are taken from ln(weight) and ln(height ^2^) in the Aurum data: ln(*a*_1_) has mean 4 and SD 0.21, ln(*a*_2_) has mean 0.97 and SD 0.11, and Corr(ln(*a*_1_),ln(*a*_2_)) = 0.22. This gives CV(*a*_2_) = 0.1.
2. Generate complete *x*_*p*_ = exp(ln(*a*_1_) − ln(*a*_2_)), meaning that *x*_*p*_ follows a lognormal distribution. For the ratios and components in our two example datasets, the lognormal distribution seems to be a suitable choice.
3. Simulate *y* ∼ N(*β*_0_ + *β*_1_*x*_*p*_,* σ*^2^). We used the same value of *β*_1_ (arbitrarily 2) throughout to make bias comparable across all simulation settings. To vary the strength of association, we altered *σ*^2^ to achieve the desired *R*^2^.
4. Simulate binary indicators of response, *R*_1_ and *R*_2_, for *a*_1_ and *a*_2_, respectively. Each *R* is generated independently from the model logit{P(*R* = 1)} = *γ*_0_ + *γ*_1_*y*. Under MCAR, *γ*_1_ = 0. Under MAR, *γ*_1_ is chosen so that ROC analysis of *y* versus an indicator of response *R* produces a mean area under the curve of 0.65. This is to achieve the same degree of MAR across scenarios. We then alter *γ*_0_ so that P(*R*_1_ = 1) = P(*R*_2_ = 1) = 0.75. Because *γ*_1_ has the same sign for both *R*_1_ and *R*_2_ and both depend on *y*, the probability of *a*_1_,*a*_2_ being missing simultaneously is slightly larger under MAR than MCAR. This means that the overall proportion of observations missing *x*_*p*_ is slightly smaller under MAR (42% missing *x*_*p*_) than MCAR (44% missing *x*_*p*_).
5. Set *a*_1*i*_ to missing if *R*_1*i*_ = 0, *a*_2*i*_ to missing if *R*_2*i*_ = 0 and *x*_*pi*_ to missing if *R*_1*i*_ = 0 or *R*_2*i*_ = 0.
6. Impute *x*_*p*_ five times using each of the models M1–M6 (Table 3).
7. Fit the correct analysis model to each imputed dataset, and combine the results using Rubin's rules.

We used 5000 replicates of this process under each combination of simulation settings. Interest is in *β*_1_. We calculated bias, coverage of 95% confidence intervals and efficiency of 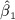 (expressed by the empirical standard error, SD 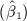 over all replications 29) under models M1–M6, with analysis of complete data (i.e. before any data are set to missing) and complete cases (dropping observations with missing *x*_*p*_) also provided for reference.

### 5.2 Results

Table 4 summarises the results of our simulation study. Results of the complete data and complete cases analyses are both as expected. Complete data are always unbiased with 95% coverage and the smallest empirical standard error of all methods. Complete cases are unbiased under MCAR but biased under MAR. Coverage is correspondingly low, and efficiency is lower than complete data.

**Table 4:** Simulation results: bias, coverage and efficiency of different imputation models.

				Bias (*β*_1_ = 2)		Empirical SE		Coverage	
*R*^2^	CV (*a*_2_)	Imputation model		MCAR	MAR	MCAR	MAR	MCAR	MAR
0.1	0.1	Complete data		0.000		0.273		95.2	
		Complete cases		0.003	− 0.172	0.366	0.352	95.1	92.6
		*x*	M1	− 0.005	− 0.004	0.368	0.386	93.8	94.9
		*x*,*a*_1_	M2	− 0.001	0.002	0.333	0.345	94.6	94.7
		*x*,*a*_2_	M3	− 0.009	− 0.003	0.363	0.383	94.6	94.9
		*x*,*a*_1_,*a*_2_	M4	− 0.005	0.005	0.330	0.342	94.7	95.0
		*a*_1_,*a*_2_	M5	− 0.017	− 0.016	0.328	0.337	94.8	95.0
		ln(*a*_1_),ln(*a*_2_)	M6	− 0.016	− 0.034	0.329	0.332	94.9	95.1
0.1	0.3	Complete data		0.006		0.267		95.3	
		Complete cases		0.001	− 0.168	0.359	0.351	95.3	92.9
		*x*	M1	− 0.009	0.005	0.358	0.385	94.7	94.9
		*x*,*a*_1_	M2	− 0.007	0.014	0.348	0.372	94.9	94.9
		*x*,*a*_2_	M3	− 0.001	0.031	0.334	0.362	95.4	95.0
		*x*,*a*_1_,*a*_2_	M4	− 0.001	0.038	0.325	0.346	95.0	94.7
		*a*_1_,*a*_2_	M5	− 0.562	− 0.665	0.350	0.334	94.3	92.6
		ln(*a*_1_),ln(*a*_2_)	M6	− 0.038	− 0.064	0.313	0.318	95.8	95.4
0.3	0.1	Complete data		0.003		0.137		95.2	
		Complete cases		0.001	− 0.139	0.183	0.188	95.5	88.5
		*x*	M1	− 0.005	0.031	0.171	0.187	95.3	94.0
		*x*,*a*_1_	M2	− 0.003	0.026	0.159	0.171	95.8	95.0
		*x*,*a*_2_	M3	− 0.007	0.029	0.170	0.188	95.2	93.8
		*x*,*a*_1_,*a*_2_	M4	− 0.003	0.026	0.159	0.171	95.9	94.6
		*a*_1_,*a*_2_	M5	− 0.016	0.000	0.158	0.168	96.1	95.3
		ln(*a*_1_),ln(*a*_2_)	M6	− 0.016	− 0.031	0.158	0.163	96.2	95.6
0.3	0.3	Complete data		− 0.002		0.137		95.0	
		Complete cases		− 0.006	− 0.143	0.184	0.192	94.9	88.5
		*x*	M1	− 0.009	0.054	0.174	0.196	94.2	93.0
		*x*,*a*_1_	M2	− 0.012	0.057	0.172	0.193	94.8	93.3
		*x*,*a*_2_	M3	− 0.010	0.076	0.170	0.191	94.3	91.5
		*x*,*a*_1_,*a*_2_	M4	− 0.009	0.080	0.167	0.187	94.2	91.8
		*a*_1_,*a*_2_	M5	− 0.580	− 0.814	0.287	0.300	94.3	93.3
		ln(*a*_1_),ln(*a*_2_)	M6	− 0.051	− 0.070	0.162	0.164	95.1	94.6

SE, standard error; CV, coefficient of variation; MCAR, missing completely at random; MAR, missing at random.

M1 is mainly unbiased, but there is a small upward bias under MAR and *R*^2^ = 0.3, and coverage is slightly low when data are MAR. This is perhaps because it assumes normality for *x*_*p*_ when it is actually lognormal. M1 also tends to be inefficient compared to other imputation models, as would be expected, regardless of the missingness mechanism.

With this general pattern of missingness, M3 is usually more biased than M2, although coverage tends to be similar (except where CV(*a*_2_) = 0.3 and *R*^2^ = 0.3). Efficiency of M2 and M3 seems to depend on CV(*a*_2_) and *R*^2^. Model M4 has similar bias to M2 and M3; at worst, this reaches about 4% with both large CV(*a*_2_) and *R*^2^. Empirical standard errors for M4 are at least as small as M2 and M3, while coverage tends to be good except when both CV(*a*_2_) and *R*^2^ are 0.3.

Model M5 performs well in the two scenarios when CV (*a*_2_) = 0.1. There is a small downward bias, but efficiency and coverage are both good compared with other methods. However, when CV(*a*_2_) = 0.3, we observe unacceptable bias towards the null and lower efficiency than other methods, although coverage is still over 90%. When considered alongside bias, this coverage implies that while the empirical standard error is large, the estimated standard errors are even larger, reducing the effect of the large bias on coverage and implying low power.

M6 is more biased than M5 when CV(*a*_2_) = 0.1 but much less so when CV(*a*_2_) = 0.3. Across all of our settings, it is more efficient than M1–M5 and with coverage close to 95%. If the small bias seems acceptable, then this is the best imputation model.

## 6 Discussion

We have presented the results of two case studies involving commonly used ratios and a simulation study based in part on these datasets. A key message is the caution against passive imputation of *a*_1_ and *a*_2_ without prior transformation. Superficially, the approach appears to make more use of the available data; however, it is often inefficient and can suffer from large bias. Our analysis of the *EPIC*-Norfolk data demonstrated this problem in practice. However, in our *Aurum* case study, the use of passive imputation appeared to make little difference to the substantive results compared to active imputation. Our simulation study confirmed that problems arise when CV(*a*_2_) is large. Note that a ratio with very small CV(*a*_2_) is unlikely to be used in applied work (unless CV(*a*_1_) is also very small) because as CV (*a*_2_) → 0, *x*_*p*_ becomes a function of *a*_1_ divided by a constant. We therefore recommend that incomplete ratios be imputed actively or passively after log transformation as in model M6.

In considering models for missing data, joint models for the covariates and outcome are attractive because they use the full data likelihood in a coherent way. In our two case studies, we attempted to fit fully Bayesian joint models and summarise posterior distributions for parameters of interest. Computational problems prevented this approach from being useful. In one dataset, some of the models did not appear to converge to any true posterior distribution (or if they did, results were extraordinarily sensitive to the choice of model for the ratio). In the other dataset, it was not possible to load the observed data into Winbugs, and so the attempt was abandoned.

Compatibility is a useful concept for considering whether various imputation models are sensible. We hypothesised that models M1 and M2–M4 would perform well because of being compatible and semi-compatible respectively, while models M5 and M6 would perform poorly because of being incompatible. In our simulations, M1–M4 did tend to perform well despite being misspecified, and model M5 did often perform poorly. In our *EPIC*-Norfolk example, where model M5 gave nonsense results, problems could be identified by inspecting the imputed values of *x*_*p*_.

Model M6 was surprisingly as good as any other model considered throughout. Despite being more robust than M5, we know it is not completely ‘safe’. In our simulation study, the imputation model assumed (log(*a*_1_),log(*a*_2_)* * | * y*) ∼ N, and because log(*x*_*p*_) = log(*a*_1_) − log(*a*_2_), this implies (log(*x*_*p*_)* * | * y*) ∼ N. The imputation model therefore has mean function log(*x*_*p*_) = *α*_0_ + *α*_1_*y*, while the analysis model has mean function *y* = *β*_0_ + *β*_1_*x*_*p*_. In further simulations, we noted that M6 was still robust when *R*^2^ = 0.5 and CV(*a*_2_) = 0.3 (results not shown). We can provide no guarantee for greater values other than that this model will eventually fall apart. However, it is our experience that associations stronger than *R*^2^ = 0.5 are rare in medical applications.

Some of the issues with model M5 could have been alleviated by using partly parametric imputation techniques such as PMM 30 or local residual draws 28. In practice, this requires a switch to the chained equations approach rather than a multivariate imputation model. Because a parametric model is used only to identify suitable donors, this makes it impossible to think about compatibility. We investigated PMM in the problematic *EPIC*-Norfolk dataset and found model M5 much improved. PMM may therefore be a useful adjunct to a suitably chosen imputation model.

In evaluating methods, we have focused on bias, coverage and efficiency. For those interested in accurate prediction, efficiency may be more important and coverage less so or even unimportant 31. It is worth noting that precision is also lower for model M5. Therefore, if passive imputation is to be used for a ratio in prediction settings, it should be performed on the log scale.

We have considered the imputation of ratio covariates. Some similar issues arise when the analysis model contains any nonlinear function, for example, interactions and squares. The difference is that in both cases, the main effects and their interaction, or the variable and its square, are included in the analysis model. In the case of squares, a measurement and its square will also be observed or missing simultaneously. Imputation is then complicated by the fact that the analysis model contains both the untransformed variable and a nonlinear function as covariates, rather than just the nonlinear function, as in the case of ratios. This makes issues around compatibility somewhat more complicated. See von Hippel 22, Seaman *et al.* 23 and Bartlett *et al.* 11 for recent work on imputation of squares and interactions.

Bartlett *et al.* proposed the use of rejection sampling when producing imputations and showed it to be useful for imputing squares and interactions; this may therefore be a good approach for imputing ratios. By explicitly involving the analysis model in the specification of the imputation model, each imputation model used in the chained equations is compatible with the imputation model 11. However, the method is more time intensive than any imputation models investigated here, and it is yet to become available in standard software packages. It also sacrifices one of the advantages of MI: separation of missing data issues from substantive analyses. However, this may be necessary and has already been partly conceded when we tailor imputation models to be compatible with the analysis model.

## Appendix A. Compatibility

Section 3.3 models are compatible if a joint model exists that implies both as conditionals. How can we tell whether there is a joint model underpinning both the imputation model and the analysis model? Arnold *et al*. give a theorem that is restated here for clarity 32.

### Theorem 1

Given two conditional densities *f*(*x * | * y*) and *g*(*y * | * x*), a joint density exists if and only if {(*x*,*y*) : *f*(*x * | * y*) > 0} = {(*x*,*y*) : *g*(*y * | * x*) > 0}, and there exist functions *u*(*x*) and *v*(*y*) such that, first,


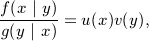
(2)

and, second, *u*(*x*) is integrable.

Here, *u*(*x*) is a marginal density for *x*, and *v*(*y*) is a marginal density for *y*. Later, we posit an analysis model and check compatibility against two different imputation models using (2).

We distinguish between two kinds of non-compatibility:

*Semi-compatibility*:  There is a special case of the imputation model that is compatible with the analysis model.
*Incompatibility*:  There is no case of the imputation model that is compatible with the analysis model.

That is, if setting certain parameters of the imputation model to 0 yields a compatible model, the imputation model is drawing on more information than the analysis model and is richer rather than the same, hence semi-compatible. If parameters of the imputation model cannot be set to 0 to identify a compatible model, the imputation model is using different information to or less information than the analysis model. Previous work has shown that incompatibility can be harmless or beneficial 18–20. When the analysis model is correctly specified, these are examples of using semi-compatible imputation models, while incompatible imputation models are always harmful when the analysis model is correctly specified.

Appendices A.1 and A.2 work through two simple examples. For both, the analysis model involves only the ratio as a covariate. Appendix A.1 uses model M5 and is shown to be incompatible; A.2 uses model M1 and is shown to be compatible.

Instead of dividing the densities, we subtract the log-densities. For clarity, we omit the intercept terms *α*_0_ and *β*_0_ from the imputation model and the analysis model, respectively, assuming both equal zero. Note that because neither parameter involves *a*_1_,*a*_2_ nor *y*, this does not impact on compatibility.

### A.1 Imputation model incompatible with the analysis model

Suppose the proposed analysis model is a linear regression of *y* on the ratio *a*_1_ / *a*_2_. The log-density for this is


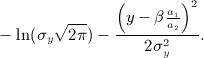
(3)

The proposed imputation model is a bivariate normal model,


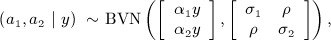


which has log-density


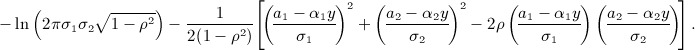
(4)

The imputation model (4) is of the form *b*(*a*_1_,*a*_2_) + *c*(*y*) + *d*_1_(*a*_1_*y*) + *d*_2_(*a*_2_*y*) and the analysis model (A.1) is of the form 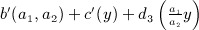. Subtracting one from the other, we cannot express the result as *u*(*a*_1_,*a*_2_) − *v*(*y*), indicating that they are incompatible.

### A.2 Imputation model compatible with the analysis model

The proposed analysis model is as in (A.1), and so the log-density is given by (3). However, the imputation model involves a linear regression of 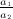 on *y*. The log-density is


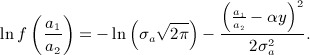
(5)

Subtracting (5) from (3), we obtain


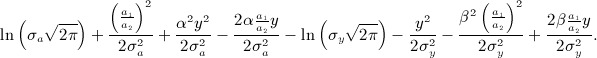
(6)

By setting 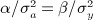, we can express (6) without any terms involving both (*a*_1_,*a*_2_) and *y*, indicating that for any choice of (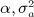), there are values of (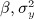) for which the proposed imputation model is compatible with the analysis model.

## Appendix B. Bayesian models for an incomplete ratio

It is conceptually natural to model missing covariates using Bayesian methods. The problem discussed in Section 3.3, that the imputation model and the analysis model may not correspond to any joint model, does not exist for Bayesian models, where the model for missing data and the analysis model are joint. The compatibility between the missing data model and the analysis model is thus assured.

The practical disadvantage of fully Bayesian models for an incomplete ratio and/or its components is computation. Bayesian models are also in general more computationally demanding than MI. Further, the imputation models described previously could be implemented fairly automatically using a choice of software, while the Bayesian models require knowledge of Winbugs 33 and/or the ability to code the models manually in another package.

Here, we explore whether Bayesian models, by working with the full joint likelihood, will provide more coherent results than MI. In our example datasets, we aim to obtain posterior means and credible intervals under various models.

### B.1 Models, software and priors

A Bayesian model combines model (1) with a model for the incomplete covariates given the complete covariates. We list candidate Bayesian models for the covariates in Table 5 (again, note the *Label* column, where the number corresponds to the imputation model with equivalent motivation). Section 3.5 and Appendix B.2 give details of how the Cox model is fit. In contrast to MI, no explicit conditioning on the outcome is required for Bayesian models.

**Table B.1:** Candidate fully Bayesian models for x_*i*_.

Model for covariates	Label
(**z**_*i*_,*x*_*pi*_* * | * ***w**_*i*_) ∼ MVN	B1
(**z**_*i*_,*x*_*pi*_,*a*_1*i*_* * | * ***w**_*i*_) ∼ MVN	B2
(**z**_*i*_,*x*_*pi*_,*a*_2*i*_* * | * ***w**_*i*_) ∼ MVN	B3
(**z**_*i*_,*x*_*pi*_,*a*_1*i*_,*a*_2*i*_* * | * ***w**_*i*_) ∼ MVN	B4
(**z**_*i*_,*a*_1*i*_,*a*_2*i*_* * | * ***w**_*i*_) ∼ MVN	B5
(**z**_*i*_,ln(*a*_1*i*_),ln(*a*_2*i*_)* * | * ***w**_*i*_) ∼ MVN	B6

Note that, except for the lack of issues around compatibility, the critique of the imputation models given in section 3.4 with equivalent labels applies equally to the Bayesian models given in Table 5. That is, models B1–B3 may ignore some of the observed data, while B2–B4 are likely to be misspecified to some degree.

To fit Bayesian joint models in our case studies, we used Winbugs 1.4.3 33. Because we are dealing with the Cox model, we used the method outlined in the Winbugs manuals (*Leuk: survival analysis using Cox regression* in Examples Volume I) to specify the models 34.

We used vague prior distributions for all parameters (see B.2 for details).

### B.2 Details on Bayesian analyses

Below, we give Winbugs code used to demonstrate the setup of the fully Bayesian Cox model where *x*_*p*_ is modelled and *a*_1_,*a*_2_ are ignored (this is the model denoted B1 in Table 3). Models B2–B6 differ only in that they simply specify the models for BMI, weight and height ^2^ differently.

The data file is made up of the covariates age sex hb logvl sqcd4 bmi, a vector of length *N* indicating death fail, a vector of length *N* of survival times for all individuals obst, and a vector of length *T* of distinct failure times t. Note that the data must be sorted in ascending order of obstbefore being passed to Winbugs. All covariates are centred at their mean.


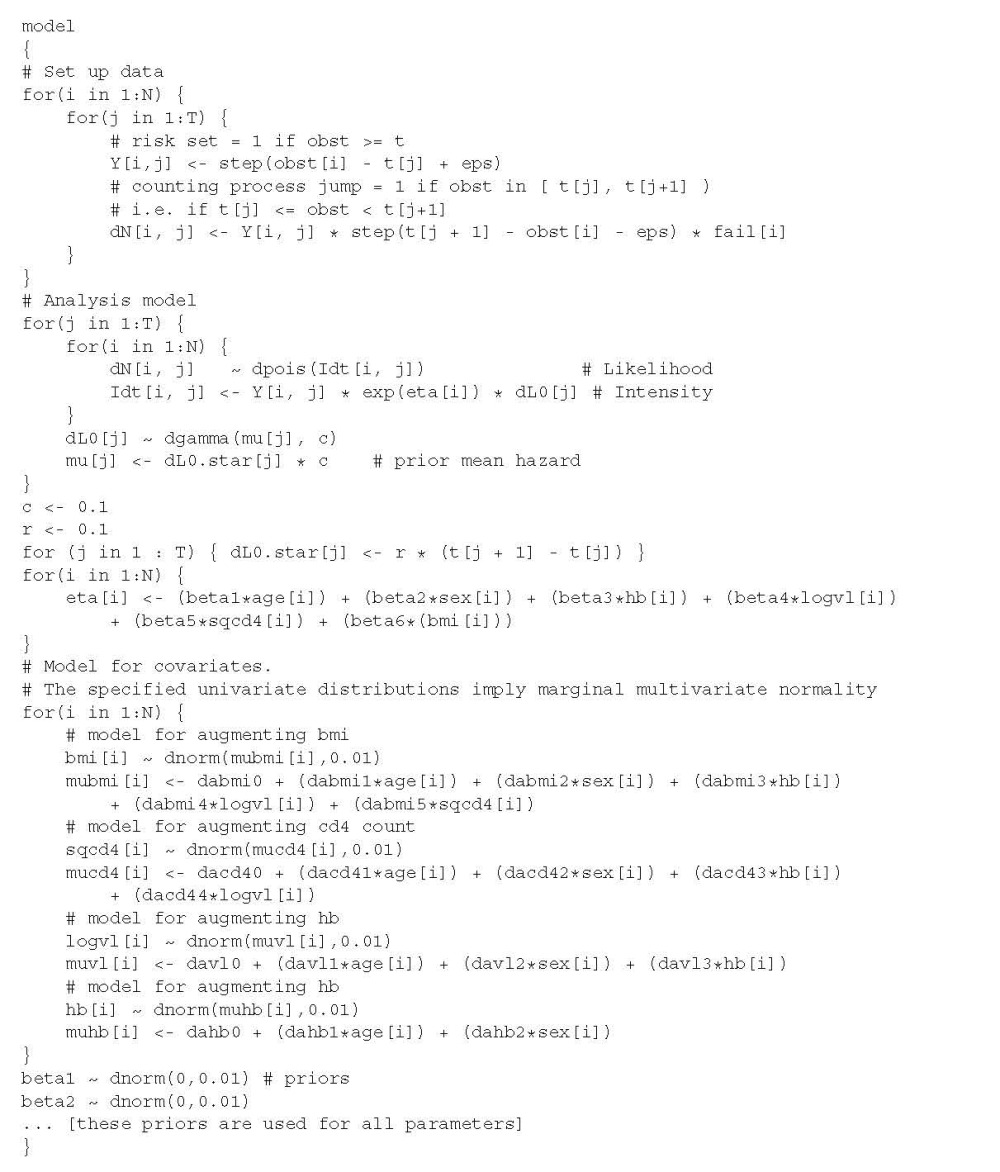


The priors for regression coefficients are ∼ N(0,100). The prior for *dL*_0_, the baseline intensity, requires slightly more explanation. This is modelled as *dL*_0_ ∼ Γ(*cr*{*t*_(*j* + 1)_ − *t*_(*j*)_},*c*), that is, a gamma distribution with mean *r*{*t*_(*j* + 1)_ − *t*_(*j*)_}and variance *r*{*t*_(*j* + 1)_ − *t*_(*j*)_} / *c*. The expression {*t*_(*j* + 1)_ − *t*_(*j*)_}is the time increment between the *j*th and *j* + 1th failure times; in the *Aurum* data, the mean time increment was 8 days. Note that *r* is not invariant to the scale of *t*, although *c* is. We used *c* = 0.1 and *r* = 0.1. A change of time scale would require *r* to be altered to obtain an equivalent prior distribution.

### B.3 Results

Fitting the Bayesian models in Winbugs was troublesome.

For the *Aurum* data, all MCMC chains ran slowly, and some stalled persistently. The simplest models (for example B1) took 5–10 h to produce 5000 iterations of the MCMC sampler. Model B5 took 10 days to produce 1000 iterations and would only update under a very specific set of initial values. Winbugs stalled repeatedly, and the need to set the model updating again inflated the run time. We present results for model B5 but do not claim the MCMC sampling converged to the true posterior distribution. Results for model B6 are absent because Winbugs was unable to sample at all; the reason for this was unclear. Winbugs ran a lot faster when fitting models that imputed missing values of *x*_*p*_ actively, that is, B1–B4.

Figure 4 presents results for the *Aurum* data (contrasting with the results obtained via MI in Figure 1). Posterior distributions obtained from different fully Bayesian analyses give diverse results. For hæmoglobin, posterior means for all models except B5 are slightly closer to 0 than any of the MI models, and the 95% credible intervals tend to be slightly shorter than the MI confidence intervals. This may in part be the effect of the prior for the hazard, as seen in the comparison of Bayesian and frequentist analysis of complete cases. Under model B5, the posterior distribution for the log hazard ratio had mean much closer to zero with smaller posterior variance than under other models.

**Figure B.1:**
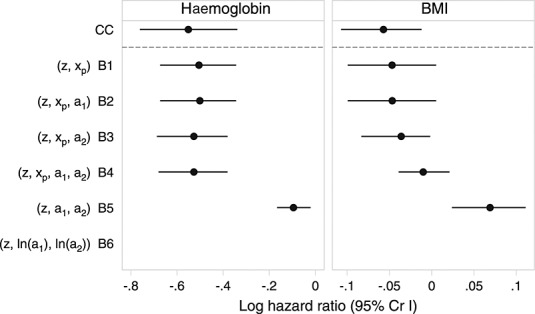
Results from analyses of *Aurum* data under different Bayesian models for body mass index (BMI).

For BMI, posterior means from B1–B5 are very variable. B1 and B2 largely agree with the MI and (Bayesian) complete cases estimates, although the intervals are longer than those obtained after MI. Posterior means from B3 and B4 are closer to 0 and have shorter credible intervals than MI models or the other Bayesian models. For B4, this perhaps reflects the incorrect assumption made about the joint distribution of *x*_*p*_,*a*_1_,*a*_2_ (this is surprising because the issue does not appear to affect model M4). Model B5 shows an effect in the *opposite* direction to all other estimates. This was the model that was very difficult to run in Winbugs. As noted previously, we do not claim B5 ever converged to the true posterior density.

For the *EPIC*-Norfolk data, it was not possible to compile any of the fully Bayesian models in Winbugs, even for complete cases. We tried compiling the complete cases model for subsets of the data of gradually increasing size (starting with *n* = 1000); model compilation failed beyond *n* > 4000. The *EPIC*-Norfolk dataset is too large for Winbugs, and so attempts to fit the fully Bayesian models were abandoned. This is a setting where a fully Bayesian analysis is impractical to any but the most dedicated.

## Appendix C. Results for *EPIC*-Norfolk after imputation using predictive mean matching

As described in Section 4.2.1, we re-ran the imputation models for *EPIC*-Norfolk using *PMM*. Figure 5 gives the full results analogous to those given in Figure 2. Note that, with the exception of model M5, there is less consistency between models than between the models that did not use PMM. Note also that the fraction of missing information is uniformly greater for the models that use PMM.

**Figure C.1:**
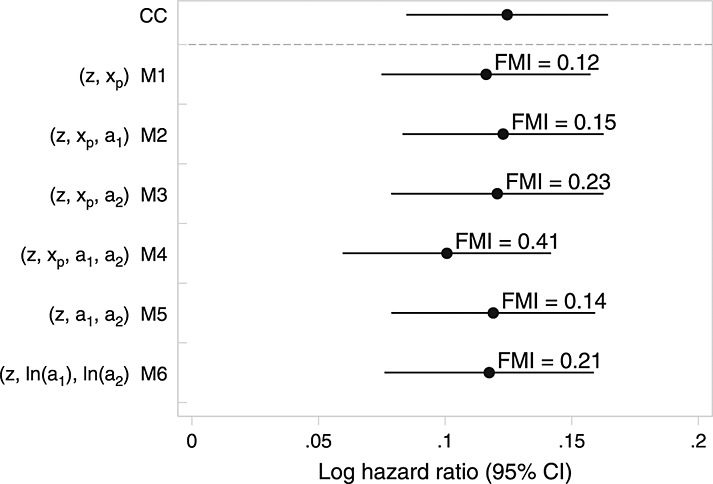
Results from analyses of *EPIC*-Norfolk data under different models for cholesterol ratio using predictive mean matching. The estimated fraction of missing information (FMI) is given next to multiple imputation analyses.
